# Author Correction: Mortality outcomes with hydroxychloroquine and chloroquine in COVID-19 from an international collaborative meta-analysis of randomized trials

**DOI:** 10.1038/s41467-024-45360-6

**Published:** 2024-02-05

**Authors:** Cathrine Axfors, Andreas M. Schmitt, Perrine Janiaud, Janneke van’t Hooft, Sherief Abd-Elsalam, Ehab F. Abdo, Benjamin S. Abella, Javed Akram, Ravi K. Amaravadi, Derek C. Angus, Yaseen M. Arabi, Shehnoor Azhar, Lindsey R. Baden, Arthur W. Baker, Leila Belkhir, Thomas Benfield, Marvin A. H. Berrevoets, Cheng-Pin Chen, Tsung-Chia Chen, Shu-Hsing Cheng, Chien-Yu Cheng, Wei-Sheng Chung, Yehuda Z. Cohen, Lisa N. Cowan, Olav Dalgard, Fernando F. de Almeida e Val, Marcus V. G. de Lacerda, Gisely C. de Melo, Lennie Derde, Vincent Dubee, Anissa Elfakir, Anthony C. Gordon, Carmen M. Hernandez-Cardenas, Thomas Hills, Andy I. M. Hoepelman, Yi-Wen Huang, Bruno Igau, Ronghua Jin, Felipe Jurado-Camacho, Khalid S. Khan, Peter G. Kremsner, Benno Kreuels, Cheng-Yu Kuo, Thuy Le, Yi-Chun Lin, Wu-Pu Lin, Tse-Hung Lin, Magnus Nakrem Lyngbakken, Colin McArthur, Bryan J. McVerry, Patricia Meza-Meneses, Wuelton M. Monteiro, Susan C. Morpeth, Ahmad Mourad, Mark J. Mulligan, Srinivas Murthy, Susanna Naggie, Shanti Narayanasamy, Alistair Nichol, Lewis A. Novack, Sean M. O’Brien, Nwora Lance Okeke, Léna Perez, Rogelio Perez-Padilla, Laurent Perrin, Arantxa Remigio-Luna, Norma E. Rivera-Martinez, Frank W. Rockhold, Sebastian Rodriguez-Llamazares, Robert Rolfe, Rossana Rosa, Helge Røsjø, Vanderson S. Sampaio, Todd B. Seto, Muhammad Shahzad, Shaimaa Soliman, Jason E. Stout, Ireri Thirion-Romero, Andrea B. Troxel, Ting-Yu Tseng, Nicholas A. Turner, Robert J. Ulrich, Stephen R. Walsh, Steve A. Webb, Jesper M. Weehuizen, Maria Velinova, Hon-Lai Wong, Rebekah Wrenn, Fernando G. Zampieri, Wu Zhong, David Moher, Steven N. Goodman, John P. A. Ioannidis, Lars G. Hemkens

**Affiliations:** 1 Meta-Research Innovation Center at Stanford (METRICS), Stanford University, Stanford, CA USA; 2 Department for Women’s and Children’s Health, Uppsala University, Uppsala, Sweden; 3 Department of Clinical Research, University Hospital Basel, University of Basel, Basel, Switzerland; 4 Department of Medical Oncology, University of Basel, Basel, Switzerland; 5 Amsterdam University Medical Center, Amsterdam University, Amsterdam, the Netherlands; 6 Tropical Medicine and Infectious Diseases Department, Faculty of Medicine, Tanta University, Tanta, Egypt; 7 Tropical Medicine and Gastroenterology Department, Faculty of Medicine, Assiut University, Assiut, Egypt; 8 Department of Emergency Medicine, University of Pennsylvania, Philadelphia, PA USA; 9 Department of Internal Medicine, Vice Chancellor, University of Health Sciences, Lahore, Punjab Pakistan; 10 Abramson Cancer Center and Department of Medicine, University of Pennsylvania, Philadelphia, PA USA; 11 Department of Critical Care Medicine, The Clinical Research Investigation and Systems Modeling of Acute Illness (CRISMA) Center, University of Pittsburgh, Pittsburgh, PA USA; 12 the UPMC Health System Office of Healthcare Innovation, University of Pittsburgh Medical Centre, Pittsburgh, PA USA; 13 Intensive Care Department, King Saud Bin Abdulaziz University for Health Sciences and King Abdullah International Medical Research Center, Riyadh, Saudi Arabia; 14 Department of Public Health, University of Health Sciences, Lahore, Punjab Pakistan; 15 Division of Infectious Diseases, Brigham and Women’s Hospital, Boston, MA USA; 16 Department of Medicine, Division of Infectious Diseases and International Health, Duke University Medical Center, Durham, NC USA; 17 Infectious Diseases Department, Cliniques universitaires Saint-Luc, Université Catholique de Louvain, Brussels, Belgium; 18 Center of Research & Disruption of Infectious Diseases, Department of Infectious Diseases, Copenhagen University Hospital, Amager and Hvidovre, Hvidovre, Denmark; 19 Department of Internal Medicine, Elisabeth-Tweesteden hospital, Tilburg, Netherlands; 20 Department of Infectious Diseases, Taoyuan General Hospital, Ministry of Health and Welfare, Taoyuan, Taiwan; 21 Department of Internal Medicine, Taichung Hospital, Ministry of Health and Welfare, Taichung, Taiwan; 22 Sanofi, Bridgewater, NJ USA; 23 Department of Infectious Diseases, Division of Medicine, Akershus University Hospital, Lørenskog, Norway; 24 Institute of Clinical Medicine, Faculty of Medicine, University of Oslo, Oslo, Norway; 25 Fundação de Medicina Tropical Dr. Heitor Vieira Dourado, Manaus, AM Brazil; 26 Instituto Leonidas e Maria Deane – ILMD, FIOCRUZ-AM, Manaus, AM Brazil; 27 Universidade do Estado do Amazonas, Manaus, AM Brazil; 28 Julius Center for Health Sciences and Primary Care, University Medical Center Utrecht, Utrecht, Netherlands; 29 Intensive Care Centre, University Medical Center Utrecht, Utrecht, Netherlands; 30 Infectious and Tropical Diseases Department, Angers University Hospital, Angers, France; 31 Ividata Life Sciences, Levallois-Perret, France; 32 Department of Surgery and Cancer, Anaesthetics, Pain Medicine, and Intensive Care Medicine, Imperial College London and Imperial College Healthcare NHS Trust, London, UK; 33 Critical Care Department, Instituto Nacional de Enfermedades Respiratorias Ismael Cosío Villegas, Ciudad de México, Mexico; 34 Medical Research Institute of New Zealand, Wellington, New Zealand; 35 Auckland City Hospital, Auckland, New Zealand; 36 Department of Infectious Diseases, University Medical Center Utrecht, Utrecht, Netherlands; 37 Department of Internal Medicine, Chang Hua Hospital, Ministry of Health and Welfare, Changhua, Taiwan; 38 Beijing Youan Hospital, Capital Medical University, Beijing, People’s Republic of China; 39 Department of Preventive Medicine & Public Health, University of Granada, Hospital Real, Avenida del Hospicio, Granada, Granada, Spain; 40 Institute of Tropical Medicine, University of Tübingen, Tübingen, Germany; 41 Centre de Recherches Médicales de Lambaréné, Lambaréné, Gabon; 42 German Center for Infection Research, Partner Site Tübingen, Tübingen, Germany; 43 Department of Medicine, Division of Tropical Medicine and Division of Infectious Diseases, University Medical Center Hamburg-Eppendorf, Hamburg, Germany; 44 Department of Tropical Medicine, Bernhard Nocht Institute for Tropical Medicine, Hamburg, Germany; 45 Department of Internal Medicine, Pingtung Hospital, Ministry of Health and Welfare, Pingtung, Taiwan; 46 Department of Internal Medicine, Taipei Hospital, Ministry of Health and Welfare, New Taipei City, Taiwan; 47 Division of Medicine, Akershus University Hospital, Lørenskog, Norway; 48 School of Epidemiology and Preventive Medicine, Australian and New Zealand Intensive Care Research Centre, Monash University, Melbourne, VIC Australia; 49 Department of Medicine, University of Pittsburgh, Pittsburgh, PA USA; 50 Hospital Regional de Alta especialidad de Ixtapaluca, Ixtapaluca, Mexico; 51 Middlemore Hospital, Auckland, New Zealand; 52 Department of Medicine, Duke University Medical Center, Durham, NC 27710 USA; 53 Department of Microbiology, NYU Grossman School of Medicine, New York, NY USA; 54 Department of Internal Medicine, Division of Infectious Diseases and Immunology, NYU Grossman School of Medicine, New York, NY USA; 55 University of British Columbia School of Medicine, Vancouver, BC Canada; 56 Department of Intensive Care, Alfred Health, Melbourne, VIC Australia; 57 Department of Anesthesia and Intensive Care, St Vincent’s University Hospital, Dublin, Ireland; 58 School of Medicine and Medical Sciences, University College Dublin, Dublin, Ireland; 59 Division of Infectious Diseases, Brigham and Women’s Hospital, Harvard Medical School, Boston, MA USA; 60 Department of Biostatistics and Bioinformatics, Duke University Medical Center and Duke Clinical Research Institute, Durham, NC USA; 61 Excelya, Montpellier, France; 62 Department of Smoking and COPD, Instituto Nacional de Enfermedades Respiratorias Ismael Cosío Villegas, Ciudad de México, Mexico; 63 Sanofi, Montpellier, France; 64 Hospital Regional de Alta especialidad de Oaxaca, Oaxaca, Mexico; 65 UnityPoint Health, Des Moines, IA USA; 66 Division of Research and Innovation, Akershus University Hospital, Lørenskog, Norway; 67 Fundação de Vigilância em Saúde do Amazonas, Manaus, AM Brazil; 68 University of Hawaii John A. Burns School of Medicine, Honolulu, HI USA; 69 The Queen’s Medical Center, Honolulu, HI USA; 70 Department of Pharmacology, University of Health Sciences, Lahore, Punjab Pakistan; 71 Public Health and Community Medicine, Menoufia University, Menoufia, Egypt; 72 Division of Biostatistics, Department of Population Health, NYU Grossman School of Medicine, New York, NY USA; 73 Department of Medicine, Division of Infectious Diseases and Immunology, NYU Grossman School of Medicine, New York, NY USA; 74 St. John of God Hospital, Subiaco, WA Australia; 75 PRA Health Science, Groningen, Netherlands; 76 Department of Internal Medicine, Keelung Hospital, Ministry of Health and Welfare, Keelung, Taiwan; 77 Research Institute, HCor-Hospital do Coração, São Paulo, Brazil; 78 Research Institute, BRICNet—Brazilian Research in Intensive Care Network, São Paulo, Brazil; 79 IDor Research Institute, São Paulo, Brazil; 80 National Engineering Research Center for the Emergency Drug, Beijing Institute of Pharmacology and Toxicology, Beijing, People’s Republic of China; 81 Centre for Journalology, Clinical Epidemiology Program, Ottawa Hospital Research Institute, Ottawa, ON Canada; 82 Stanford University School of Medicine, Stanford, CA USA; 83 Department of Epidemiology and Population Health, Stanford University School of Medicine, Stanford, CA USA; 84 Stanford Prevention Research Center, Department of Medicine, Stanford University, Stanford, CA USA; 85 Meta-Research Innovation Center Berlin (METRIC-B), Berlin Institute of Health, Berlin, Germany

**Keywords:** Viral infection, Epidemiology

Correction to: *Nature Communications* 10.1038/s41467-021-22446-z, published online 15 April 2021

The original version of this article contained an error in Table 1, which misidentified the trial included in the meta-analysis registered as NCT04323527 as CloroCOVID19II instead of CloroCOVID19III. The NCT04323527 registration includes the trials CloroCOVID19I and CloroCOVID19III. CloroCOVID19I was not included in the meta-analysis.

In addition, the original version of the Methods section inadvertently omitted details of which formulations of hydroxychloroquine or chloroquine the reported dosages refer to. The following information has been included in the legend for Table 1 and in the corrected methods section: “In all trials that used hydroxychloroquine, dosages refer to hydroxychloroquine sulfate. In trials that used chloroquine, the dosages for ARCHAIC, ChiCTR2000030054 and ChiCTR2000031204 refer to chloroquine phosphate, while those for CloroCOVID19II and CloroCOVID19III refer to chloroquine base.

The American Journal of Tropical Medicine and Hygiene has issued a retraction note (1) for one of the trials (2) that had been included in the calculations of our meta-analysis. Exclusion of the data from this trial changes neither the results nor inferences of the meta-analysis. For hydroxychloroquine, the original odds ratio for mortality was 1.11 (95% CI: 1.02–1.20; *I*^2^ = 0%; 26 trials; 10,012 patients) and excluding the retracted trial the odds ratio for mortality would remain 1.11 (95% CI: 1.02–1.20, *I*^2^ = 0%; 25 trials; 9818 patients).

1. Retraction Notice. The American Journal of Tropical Medicine and Hygiene 107, 728-728, 10.4269/ajtmh.1073ret (2022).
2. Abd-Elsalam, S. et al. RETRACTED: Hydroxychloroquine in the Treatment of COVID-19: A Multicenter Randomized Controlled Study. Am J Trop Med Hyg 103, 1635-1639, 10.4269/ajtmh.20-0873 (2020).

The errors in Table 1 and in the Methods section have been corrected in both the PDF and HTML versions of the Article.

