# Supplementary material for: Mortality outcomes with hydroxychloroquine and chloroquine in COVID-19 from an international collaborative meta-analysis of randomized trials
Source: Nat Commun. 2021 Apr 15;12:2349. doi: 10.1038/s41467-021-22446-z (PMC8050319; doi:10.1038/s41467-021-22446-z)
Supplement: Supplementary file 1 — Supplementary Information [file 41467_2021_22446_MOESM1_ESM.pdf]

Supplements to "Mortality outcomes with hydroxychloroquine and chloroquine in COVID-19 from an international collaborative meta-analysis of randomized trials"

1. Supplementary Note 1 - PRISMA Checklist
2. Supplementary Note 2 - Search strategy
3. Supplementary Note 3- Template for invitation email
4. Table S1. Group-level characteristics of unavailable trials
5. Table S2. Risk of bias assessment
6. Table S3. Subgroup analyses
7. Table S4. Sensitivity analyses
8. Figure S1. Funnel plot
9. Figure S2A-B. Forest plots for subgroup analyses
10. Figure S3A-C. Forest plots for sensitivity analyses

Supplementary Note 1 – PRISMA checklist

Section/topic	#	Checklist item	Reported on page #
**TITLE**			
Title	1	Identify the report as a systematic review, meta-analysis, or both.	1
**ABSTRACT**			
Structured summary	2	Provide a structured summary including, as applicable: background; objectives; data sources; study eligibility criteria, participants, and interventions; study appraisal and synthesis methods; results; limitations; conclusions and implications of key findings; systematic review registration number.	6
**INTRODUCTION**			
Rationale	3	Describe the rationale for the review in the context of what is already known.	7
Objectives	4	Provide an explicit statement of questions being addressed with reference to participants, interventions, comparisons, outcomes, and study design (PICOS).	8
**METHODS**			
Protocol and registration	5	Indicate if a review protocol exists, if and where it can be accessed (e.g., Web address), and, if available, provide registration information including registration number.	14
Eligibility criteria	6	Specify study characteristics (e.g., PICOS, length of follow-up) and report characteristics (e.g., years considered, language, publication status) used as criteria for eligibility, giving rationale.	14
Information sources	7	Describe all information sources (e.g., databases with dates of coverage, contact with study authors to identify additional studies) in the search and date last searched.	14
Search	8	Present full electronic search strategy for at least one database, including any limits used, such that it could be repeated.	Supplement Note 2
Study selection	9	State the process for selecting studies (i.e., screening, eligibility, included in systematic review, and, if applicable, included in the meta-analysis).	14-15
Data collection process	10	Describe method of data extraction from reports (e.g., piloted forms, independently, in duplicate) and any processes for obtaining and confirming data from investigators.	15

Data items	11	List and define all variables for which data were sought (e.g., PICOS, funding sources) and any assumptions and simplifications made.	15
Risk of bias in individual studies	12	Describe methods used for assessing risk of bias of individual studies (including specification of whether this was done at the study or outcome level), and how this information is to be used in any data synthesis.	15
Summary measures	13	State the principal summary measures (e.g., risk ratio, difference in means).	16
Synthesis of results	14	Describe the methods of handling data and combining results of studies, if done, including measures of consistency (e.g., I^2) for each meta-analysis.	16

Section/topic	#	Checklist item	Reported on page #
Risk of bias across studies	15	Specify any assessment of risk of bias that may affect the cumulative evidence (e.g., publication bias, selective reporting within studies).	15
Additional analyses	16	Describe methods of additional analyses (e.g., sensitivity or subgroup analyses, meta-regression), if done, indicating which were pre-specified.	16-17
**RESULTS**			
Study selection	17	Give numbers of studies screened, assessed for eligibility, and included in the review, with reasons for exclusions at each stage, ideally with a flow diagram.	9, Figure 1
Study characteristics	18	For each study, present characteristics for which data were extracted (e.g., study size, PICOS, follow-up period) and provide the citations.	9
Risk of bias within studies	19	Present data on risk of bias of each study and, if available, any outcome level assessment (see item 12).	9-10 Supplement
Results of individual studies	20	For all outcomes considered (benefits or harms), present, for each study: (a) simple summary data for each intervention group (b) effect estimates and confidence intervals, ideally with a forest plot.	Figure 2 & 3
Synthesis of results	21	Present results of each meta-analysis done, including confidence intervals and measures of consistency.	10
Risk of bias across studies	22	Present results of any assessment of risk of bias across studies (see Item 15).	9-10, Supplement
Additional analysis	23	Give results of additional analyses, if done (e.g., sensitivity or subgroup analyses, meta-regression [see Item 16]).	10
**DISCUSSION**			

Summary of evidence	24	Summarize the main findings including the strength of evidence for each main outcome; consider their relevance to key groups (e.g., healthcare providers, users, and policy makers).	11
Limitations	25	Discuss limitations at study and outcome level (e.g., risk of bias), and at review-level (e.g., incomplete retrieval of identified research, reporting bias).	12-13
Conclusions	26	Provide a general interpretation of the results in the context of other evidence, and implications for future research.	13
**FUNDING**			
Funding	27	Describe sources of funding for the systematic review and other support (e.g., supply of data); role of funders for the systematic review.	22-23

From: Moher D, Liberati A, Tetzlaff J, Altman DG, The PRISMA Group (2009). Preferred Reporting Items for Systematic Reviews and Meta-Analyses: The PRISMA Statement. PLoS Med 6(7): e1000097. doi:10.1371/journal.pmed1000097

For more information, visit: **www.prisma-statement.org**.

Supplementary Note 2 - Search strategy

The COVID-evidence database includes trials registered on ClinicalTrials.gov or the WHO International Clinical Trials Registry Platform up to June 11, 2020, as well as trials published on the following sources up to April 9, 2020: PubMed, medrXiv, biorXiv, the WHO COVID-19 literature database, and a listing of all trials with ethical approval in Switzerland (for details please see the COVID-evidence study protocol on the Open Science Framework: http://dx.doi.org/10.17605/OSF.IO/GEHFX).
This Supplement describes the search strategy used to complement the COVID-evidence database with trials registered or published after April 9, 2020.

PubMed and the Cochrane COVID-19 trial registry were searched from inception to June 11, 2020. Search terms included extensive controlled vocabulary and Medical Subject Headings (MeSH). Search terms were the following:
corona[ti] OR covid*[ti] OR sars[ti] OR severe acute respiratory syndrome[ti] OR ncov*[ti] OR "severe acute respiratory syndrome coronavirus 2" [Supplementary Concept] OR "COVID-19" [Supplementary Concept] OR (wuhan[tiab] AND coronavirus[tiab]) OR (wuhan[tiab] AND pneumonia virus[tiab]) OR COVID19[tiab] OR COVID-19[tiab] OR coronavirus 2019[tiab] OR SARS-CoV-2[tiab] OR SARS2[tiab] OR SARS-2[tiab] OR "severe acute respiratory syndrome 2"[tiab] OR 2019-nCoV[tiab] OR (novel coronavirus[tiab] AND 2019[tiab]) NOT (animals[mesh] NOT humans[mesh]) AND ("2019/12/01"[EDAT] : "3000/12/31"[EDAT])
AND
(((hydroxychloroquine[MeSH Terms]) OR (chloroquine[MeSH Terms])) OR (hydroxychloroquine[Title/Abstract])) OR (chloroquine[Title/Abstract])
AND
(randomized controlled trial[pt] OR controlled clinical trial[pt] OR randomized[tiab] OR placebo[tiab] OR clinical trials as topic[mesh:noexp] OR randomly[tiab] OR trial[ti] NOT (animals[mh] NOT humans [mh]))

The search was updated on October 16, 2020.

Supplementary Note 3 - Template for invitation email

Subject: Invitation to co-author a large-scale international collaborative meta-analysis on mortality in COVID-19 trials

Dear Dr. <last name>,

We are currently conducting a large-scale international collaborative meta-analysis on mortality in all ongoing or completed randomized clinical trials evaluating hydroxychloroquine or chloroquine for COVID-19. We are inviting all research groups worldwide testing these drugs to provide urgently needed evidence. We have no commercial interest with this work and aim to rapidly publish the results in a peer-reviewed journal. Your core team is invited to co-author the publication.
We use our COVID-evidence database (www.covid-evidence.org) for this work. COVID-evidence is supported by the Swiss National Science Foundation (Project ID 196190) and a large collaboration of researchers from Switzerland, the US, China, Canada, UK, France, Germany, Austria, Sweden, Netherlands, and other countries. Our registered protocol can be found attached as well as registered on the Open Science Framework: [link]. Trials that are eligible for this project can be found at the end of the protocol.

Your study <url> is of high importance for this project. We would like to ask you how many patients died in your trial (see short questions below). We will use standard methods of meta-analysis and focus only on group-level (aggregated) mortality (no individual patient data needed). We will not do an in-depth review of the included trials as we aim to rapidly provide results, ideally from all trials worldwide. We describe the details of the study in the attached protocol.

For the meta-analysis, we kindly ask you to answer the following questions before July 7. If you are interested in collaborating, please let us know of your interest as soon as possible.

Question 1: Could you please confirm that these criteria apply to your trial?
a) The trial is randomized and started enrollment before June 1, 2020
b) The trial has at least one group of patients who receive hydroxychloroquine or chloroquine
c) The trial has at least one control group that does not receive hydroxychloroquine or chloroquine

Question 2: For each of your study arms,
a) What intervention did this group receive?
b) How many patients were randomized to this group?
c) Of these patients, how many have died?
d) Of these patients, for how many it is unknown if they are dead or alive?

Please note that we are interested in these raw numbers regardless of the results of any statistical test. The numbers will be used to finalize the manuscript described in our registered protocol (attached).

We strive for a rapidly available, maximally informative publication with full transparency on methods. With this publication we aim to make sure that all clinical trial data hitherto collected (unpublished or published) will be of use, regardless of whether the target sample size of each trial was reached or not. We invite your core investigator team as co-authors. The manuscript will be shared with all co-authors for comments and the finalized manuscript will be uploaded as a preprint at medrXiv in parallel with submission to a peer-reviewed medical journal such as JAMA or the BMJ.

Thank you for considering our request! We kindly ask for your answer before July 7. If you are interested in collaborating, but are uncertain whether the data may be shared before July 7, please respond as soon as possible.
Should you have any questions or comments, please let us know.

Best regards,
Cathrine Axfors, Andreas Schmitt, David Moher, Steve Goodman, John Ioannidis and Lars Hemkens for the COVID-evidence team
www.covid-evidence.org

Table S1. Group-level characteristics of randomized clinical trials evaluating hydroxychloroquine or chloroquine as treatment for COVID-19 not included in the meta-analysis.

Register ID	Contact name	Arms (n)	Treatment comparisons		Targeted sample size	Location	Setting	Trial status
			Experimental group (HCQ or CQ)	Control group				
NCT04315948	Ader	4	HCQ	SoC	3100	International	Inpatient	Recruiting
NCT04391127	Arreola Guerra	5	HCQ	Placebo	200	Mexico	Inpatient	Active, not recruiting
NCT04351516	Bitzer	2	HCQ	Placebo	350	Germany	Outpatient	Recruiting
NCT04359953	Blanc	4	HCQ	Soc	1600	France	Inpatient	Recruiting
ISRCTN86534580	Butler	2	HCQ	SoC	3000	Europe	Inpatient	Recruiting
ChiCTR2000029939	Cai	2	CQ	SoC	100	China	Inpatient	Recruiting
EUCTR2020-001270-29	Sanofi Aventis study team	2	HCQ	SoC	350	International	Inpatient	Terminated
ChiCTR2000030031	Deng	2	CQ	Placebo	120	China	Inpatient	Discontinued
NCT04331600	Duda-Sikula	2	CQ	SoC	400	Poland	Inpatient	Recruiting
NCT04328272	Farooq	3	HCQ	Placebo	75	Asia	Unclear	Not yet recruiting
NCT04339816	František	3	HCQ	Placebo	240	Europe	Inpatient	Recruiting
NCT04353037	Griffin	4	HCQ	Placebo	850	United States	Outpatient	Recruiting
NCT04349592	Harris	3	HCQ + Placebo	Placebo	456	Qatar	Inpatient	Recruiting
ChiCTR2000029761	Huang	4	HCQ (3 arms with 3 different dosis)	SoC	240	China	Inpatient	Discontinued
ChiCTR2000029762	Huang	2	HCQ	SoC	60	China	Inpatient	Discontinued
EUCTR 2020-001469-35	Jankowska	2	CQ + telemedicine	telemedicine	400	Poland	Outpatient	Recruiting
NCT04354428	Johnston	3	HCQ + Placebo	Placebo + Placebo	630	United States	Outpatient	Recruiting
NCT04307693	Kim	3	HCQ	SoC	150	Asia	Inpatient	Terminated
NCT04394442	Lutfy	2	HCQ	SoC	200	Saudi Arabia	Unclear	Recruiting
ChiCTR2000029837	Mao	2	CQ	Placebo	120	China	Inpatient	Discontinued
ChiCTR2000029826	Mao	2	CQ	Placebo	45	China	Inpatient	Discontinued
EUCTR 2020-001587-29	Menéndez	2	HCQ	Placebo	714	Spain	Inpatient	Recruiting
NCT04329611	Metz	2	HCQ	Placebo	1660	Canada	Inpatient	Terminated
NCT04332991	Oldmixon	2	HCQ	Placebo	510	United States	Inpatient	Completed

NCT04342169	Pacchia	2	HCQ	Placebo	400	United States	Outpatient	Recruiting
NCT04353271	Richards	2	HCQ	Placebo	58	United States	Outpatient	Terminated
NCT04351191	Sarwar	4	HCQ (2arms with different dosis)	Placebo	400	Pakistan	Unclear	Recruiting
			CQ	Placebo				
NCT04346667	Sarwar	4	HCQ (2 arms with different dosis)	Placebo	400	Pakistan	Outpatient	Recruiting
			CQ	Placebo				
NCT04330586	Song	3	HCQ + Ciclesonide	Ciclesonide	141	Korea	Inpatient	Recruiting
ChiCTR2000030987	Tong	3	CQ + Favipiravir	Favipiravir	150	China	Inpatient	Recruiting
EUCTR 2020-001265-36	UCD study team	3	HCQ	SoC	267	Ireland	Unclear	Terminated
ChiCTR2000030417	Xu	2	CQ	Placebo	30	China	Inpatient	Discontinued
ChiCTR2000029740	Zhang	2	HCQ	SoC	78	China	Inpatient	Recruiting
ChiCTR2000029992	Zhenyu	3	HCQ	SoC	100	China	Inpatient	Not yet recruiting
			CQ	SoC				

* Trial includes more treatment arms than reported here; target sample size refers to all arms. Trial marked as "International" involves centers in multiple countries.

CQ Chloroquine, HCQ Hydroxychloroquine, SoC standard of care.

Table S2. Risk of bias, according to the revised risk-of-bias tool for randomized trials (RoB 2.0)

Acronym / Registration ID	Randomization Process	Deviations from the intended interventions	Missing Outcome Data	Measurement of the Outcome	Selection of the reported result	Overall
NCT04353336	some concerns*	low risk	low risk	low risk	low risk	some concerns
PATCH	low risk	low risk	low risk	low risk	low risk	low risk
PROTECT	low risk	low risk	low risk	low risk	low risk	low risk
CCAP-1	low risk	low risk	low risk	low risk	low risk	low risk
COVID-PEP	low risk	low risk	low risk	low risk	low risk	low risk
NCT04384380	low risk	low risk	low risk	low risk	low risk	low risk
NCT04333654	low risk	low risk	low risk	low risk	low risk	low risk
NO COVID-19	low risk	low risk	low risk	low risk	low risk	low risk
Hycovid	low risk	low risk	low risk	low risk	low risk	low risk
COV-HCQ	low risk	low risk	low risk	low risk	low risk	low risk
COMIHY	low risk	low risk	low risk	low risk	low risk	low risk
RECOVERY	low risk	low risk	low risk	low risk	low risk	low risk
CloroCOVID19II	low risk	low risk	low risk	low risk	low risk	low risk
REMAP-CAP	low risk	low risk	low risk	low risk	low risk	low risk
ChiCTR2000029868	low risk	low risk	low risk	low risk	low risk	low risk
HYDRA	low risk	low risk	low risk	low risk	low risk	low risk
ChiCTR2000031204	some concerns*	low risk	low risk	low risk	low risk	some concerns

NCT04491994	low risk	low risk	low risk	low risk	low risk	low risk
WHO SOLIDARITY	low risk	low risk	low risk	low risk	low risk	low risk
NCT04261517	some concerns*	low risk	low risk	low risk	low risk	some concerns
BCN PEP CoV-2 Study	low risk	low risk	low risk	low risk	low risk	low risk
ARCHAIC	high risk**	low risk	low risk	low risk	low risk	high risk
TEACH	low risk	low risk	some concerns***	low risk	low risk	some concerns
OAHU-COVID19	low risk	low risk	low risk	low risk	low risk	low risk
NCT04335552	some concerns*	low risk	low risk	low risk	low risk	some concerns
ChiCTR2000030054	low risk	low risk	low risk	low risk	low risk	low risk
Coalition I	low risk	low risk	low risk	low risk	low risk	low risk
ChiCTR2000029559	low risk	low risk	low risk	low risk	low risk	low risk

* Some concerns due to missing information regarding the allocation concealment. ** High risk because of predictable randomization sequence. *** The proportion of patients lost to follow-up for phone visits at day 30 was similar in the treatment and control groups (14/67 and 11/61, respectively).

Table S3. Subgroup analyses for random-effects meta-analysis on mortality for treatment of COVID-19 with Hydroxychloroquine.

Subgroup	Trials (n)	Patients (n)	OR (95% CI)	P value, test for interaction
Setting				
ICU	1	142	1.04 (0.49, 2.18)	0.98
Inpatient	21	9062	1.11 (1.02, 1.21)	
Outpatient	4	808	1.01 (0.06, 16.28)	
Published				
Yes	14	8981	1.12 (1.08, 1.16)	0.23
No	12	1031	0.92 (0.63, 1.34)	
Control				
Standard of care	17	8911	1.12 (1.04, 1.21)	0.15
Placebo	9	1101	0.88 (0.55, 1.41)	
COVID-19 diagnostic confirmation				
Confirmed	22	4215	1.11 (0.91, 1.36)	0.94
Confirmed or suspected	4	5797	1.1 (1.07, 1.14)	
Dose*				
High	3	6711	1.12 (1.01, 1.25)	0.29
Low	23	3301	0.97 (0.73, 1.30)	
Blinding				
Double	10	1163	0.88 (0.55, 1.41)	0.09
Investigator	1	373	0.47 (0.00, 377.44)	
None	15	8476	1.12 (1.04, 1.22)	

* High dose: ≥1600 mg on day 1 and ≥800 mg from day 2. Low dose: <1600 mg on day 1 or <800 from day 2.

OR odds ratio, CI confidence interval, ICU intensive care unit.

Table S4. Sensitivity analysis for meta-analysis for mortality for treatment of COVID-19 with Hydroxychloroquine using different methods of combination.

	Reciprocal of contrasting arm added to zero events	Excluding trials with zero deaths in one or both arms	Trials with more than 50 participants*	Arcsine difference
	n = 26	n = 12	n = 15	n = 26
	OR (95% CI), I^2, Tau^2			ASD (95% CI), I^2, Tau^2
HKSJ-PM	1.11 (1.02, 1.20); 0,0	1.10 (1.03, 1.18); 0,0	1.10 (1.02, 1.19); 0,0	0.01 (0, 0.03); 0,0
HKSJ-SJ	1.06 (0.80, 1.40); 0,0.44	1.08 (0.96, 1.21); 0,0.02	1.04 (0.88, 1.22); 0,0.09	0 (-0.02, 0.03); 0,0
MH-DSL	1.11 (0.98, 1.24); 0,0	1.10 (0.98, 1.24); 0,0	1.10 (0.98, 1.24); 0,0	0.01 (-0.01, 0.03); 0,0
Peto	1.11 (0.98, 1.25); 0,0	1.10 (0.98, 1.24); 0,0	1.10 (0.98, 1.24); 0,0	0.01 (-0.01, 0.03); 0,0

HKSJ-PM = Hartung-Knapp-Sidik-Jonkman adjustment for random effects model, Paule-Mandel estimator for tau^2;
HKSJ-SJ = Hartung-Knapp-Sidik-Jonkman adjustment for random effects model, Sidik-Jonkman estimator for tau^2;
MH-DSL = Mantel-Haenszel method for random effects model, DerSimonian-Laird estimator for tau^2;
PETO = Peto method with random effects model, Paule-Mandel estimator for tau^2;

* Zero events were corrected by adding the reciprocal of the size of the contrasting study arm

Figure S1 Funnel plot

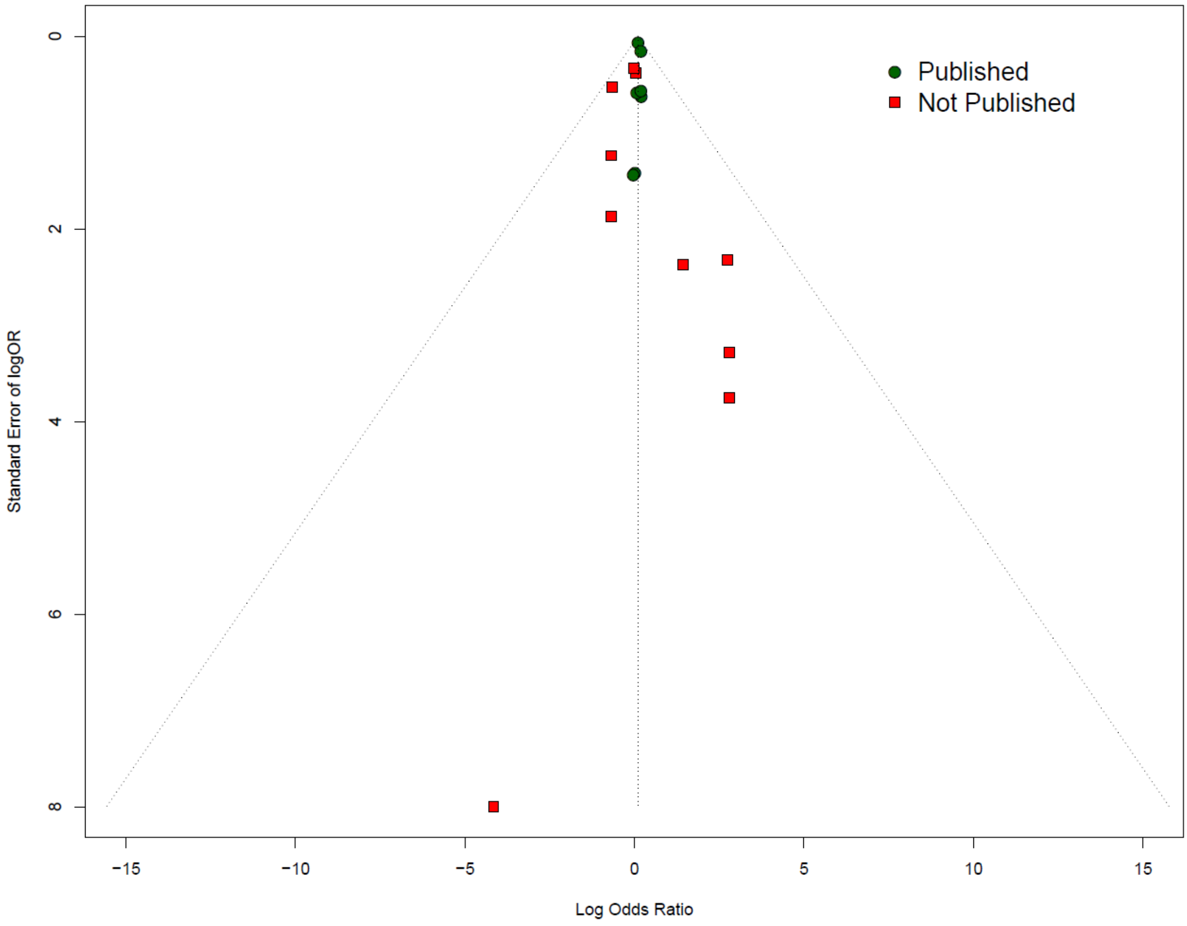

Egger’s test p value = 0.88.
The dashed vertical line denotes the log of the overall odds ratio of 1.11.

Figure S2A. Random effects meta-analysis for mortality for treatment of COVID-19 with Hydroxychloroquine, trials are stratified by control type.

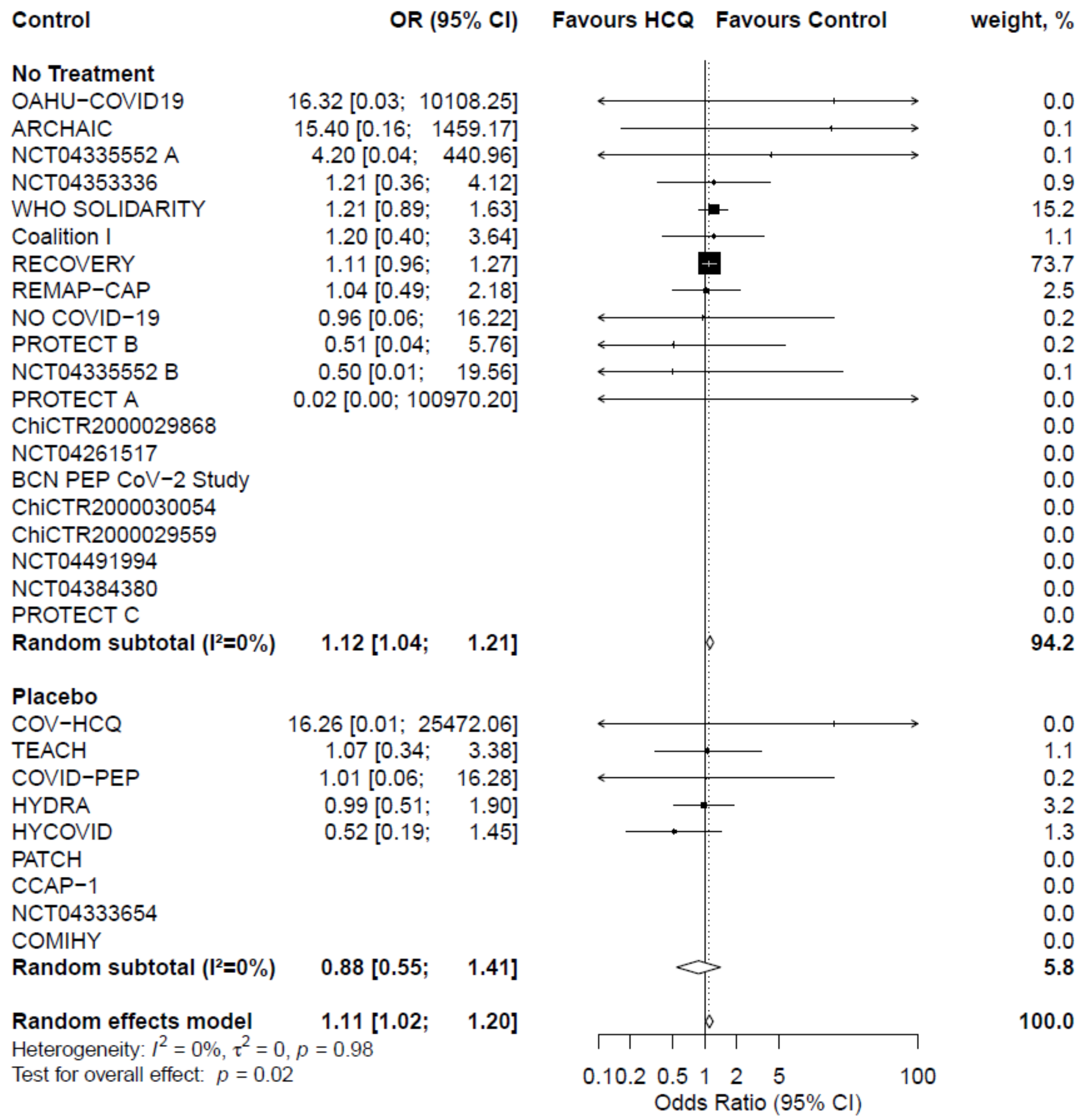

Hydroxychloroquine (HCQ) was evaluated in 17 trials with no treatment in the control group with overall 8911 patients. In these trials, 3764 patients were treated with HCQ, of whom 563 died. 5147 patients were allocated to the control group of which 913 died. Nine trials used placebo in the control group, with overall 1101 patients. In these trials, 552 patients were treated with HCQ of whom 43 died. 549 were allocated to the control group using placebo of whom 47 died. The dashed vertical line denotes an odds ratio of 1.0, which represents no difference in risk between HCQ and the control. The black horizontal bars represent 95% confidence intervals (CI). Random-effects model of the Hartung-Knapp-Sidik-Jonkman approach was performed to obtain a pooled estimate of the odds ratio. The

estimate of heterogeneity (τ^2) was obtained using the Paule and Mandel (PM) estimator. We describe the between-trial heterogeneity using the I^2-statistic. The results of the statistical tests for the overall effect and corresponding p-values are presented. All tests were two-tailed.

Figure S2B. Random effects meta-analysis for mortality for treatment of COVID-19 with Hydroxychloroquine, trials are stratified by treatment setting.

Treatment Setting	OR (95% CI)	weight, %
**Inpatient**		
OAHU−COVID19	16.32 [0.03; 10108.25]	0.0
COV−HCQ	16.26 [0.01; 25472.06]	0.0
ARCHAIC	15.40 [0.16; 1459.17]	0.1
NCT04335552 A	4.20 [0.04; 440.96]	0.1
NCT04353336	1.21 [0.36; 4.12]	0.9
WHO SOLIDARITY	1.21 [0.89; 1.63]	15.2
Coalition I	1.20 [0.40; 3.64]	1.1
RECOVERY	1.11 [0.96; 1.27]	73.7
TEACH	1.07 [0.34; 3.38]	1.1
HYDRA	0.99 [0.51; 1.90]	3.2
NO COVID−19	0.96 [0.06; 16.22]	0.2
HYCOVID	0.52 [0.19; 1.45]	1.3
PROTECT B	0.51 [0.04; 5.76]	0.2
NCT04335552 B	0.50 [0.01; 19.56]	0.1
PROTECT A	0.02 [0.00; 100970.20]	0.0
ChiCTR2000029868		0.0
NCT04261517		0.0
ChiCTR2000030054		0.0
ChiCTR2000029559		0.0
NCT04491994		0.0
NCT04384380		0.0
PATCH		0.0
PROTECT C		0.0
CCAP−1		0.0
**Random subtotal (I²=0%)**	**1.11 [1.02; 1.21]**	**97.3**
**Outpatient**		
COVID−PEP	1.01 [0.06; 16.28]	0.2
BCN PEP CoV−2 Study		0.0
NCT04333654		0.0
COMIHY		0.0
**Random subtotal (I²=0%)**	**1.01 [0.06; 16.28]**	**0.2**
**ICU**		
REMAP−CAP	1.04 [0.49; 2.18]	2.5
**Random subtotal (I²=0%)**	**1.04 [0.49; 2.18]**	**2.5**
**Random effects model**	**1.11 [1.02; 1.20]**	**100.0**

Heterogeneity: $I^2 = 0\%$, $\tau^2 = 0$, $p = 0.98$
Test for overall effect: $p = 0.02$

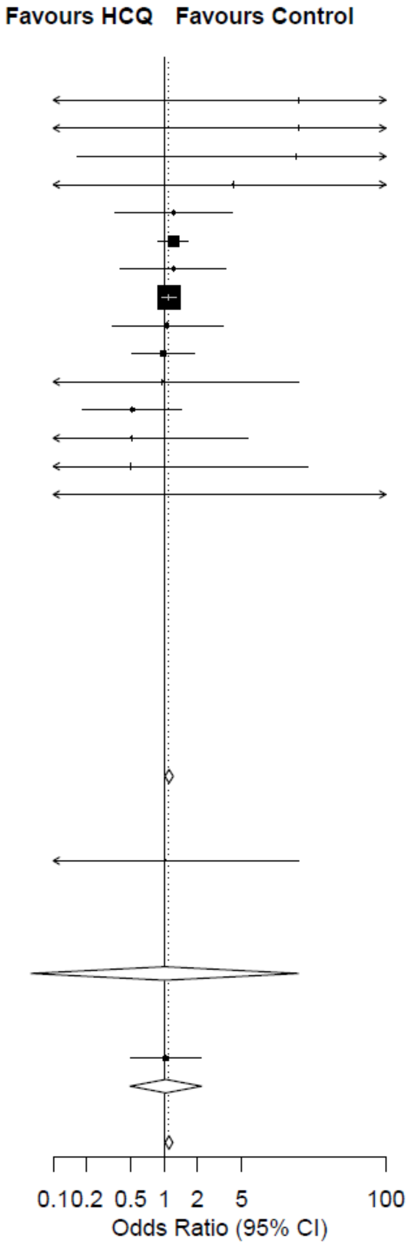

HCQ was evaluated in 21 trials with patients in an inpatient setting with overall 9062 patients. In these trials, 3862 patients were treated with HCQ, of whom 588 died. 5200 patients were allocated to the control group of which 937 died. Four trials were conducted in an outpatient setting with overall 808 patients. In these trials, 393 patients were treated with HCQ of whom 1 died. 415 were allocated to the control group of whom 1 died. One trial was conducted with patients treated on an ICU with overall 142 patients. In this trial, 61 patients were treated with HCQ, 17 of those patients died. 81 patients were allocated to the control group, 22 of those patients died. The dashed vertical line

denotes an odds ratio of 1.0, which represents no difference in risk between HCQ and the control. The black horizontal bars represent 95% confidence intervals (CI). Random-effects model of the Hartung-Knapp-Sidik-Jonkman approach was performed to obtain a pooled estimate of the odds ratio. The estimate of heterogeneity (τ^2) was obtained using the Paule and Mandel (PM) estimator. We describe the between-trial heterogeneity using the I^2-statistic. The results of the statistical tests for overall effect and corresponding p-values are presented. All tests were two-tailed.

Figure S3A. Forest plot of HKSJ-SJ model. Zero events were corrected by adding the reciprocal of the size of the contrasting study arm.

Source	OR (95% CI)	Weight, %
**Published***		
NCT04353336	1.21 [0.36; 4.12]	8.0
WHO SOLIDARITY	1.21 [0.89; 1.63]	14.1
Coalition I	1.20 [0.40; 3.64]	8.7
RECOVERY	1.11 [0.96; 1.27]	14.7
TEACH	1.07 [0.34; 3.38]	8.4
COVID−PEP	1.01 [0.06; 16.28]	2.7
NO COVID−19	0.96 [0.06; 16.22]	2.6
ChiCTR2000029868		0.0
NCT04261517		0.0
BCN PEP CoV−2 Study		0.0
ChiCTR2000030054		0.0
ChiCTR2000029559		0.0
NCT04491994		0.0
NCT04384380		0.0
**Random subtotal (I^2=0%)**	**1.12 [1.08; 1.17]**	**59.2**
**Not Published**		
OAHU−COVID19	16.32 [0.03; 10108.25]	0.6
COV−HCQ	16.26 [0.01; 25472.06]	0.5
ARCHAIC	15.40 [0.16; 1459.17]	1.1
NCT04335552 A	4.20 [0.04; 440.96]	1.1
REMAP−CAP	1.04 [0.49; 2.18]	11.3
HYDRA	0.99 [0.51; 1.90]	11.9
HYCOVID	0.52 [0.19; 1.45]	9.2
PROTECT B	0.51 [0.04; 5.76]	3.3
NCT04335552 B	0.50 [0.01; 19.56]	1.7
PROTECT A	0.02 [0.00; 100970.20]	0.1
PATCH		0.0
PROTECT C		0.0
CCAP−1		0.0
NCT04333654		0.0
COMIHY		0.0
**Random subtotal (I^2=0%)**	**1.04 [0.48; 2.21]**	**40.8**
**Random effects model**	**1.06 [0.80; 1.40]**	**100.0**

Heterogeneity: I^2 = 0%, τ^2 = 0.4444, p = 0.98
Test for overall effect: p = 0.67

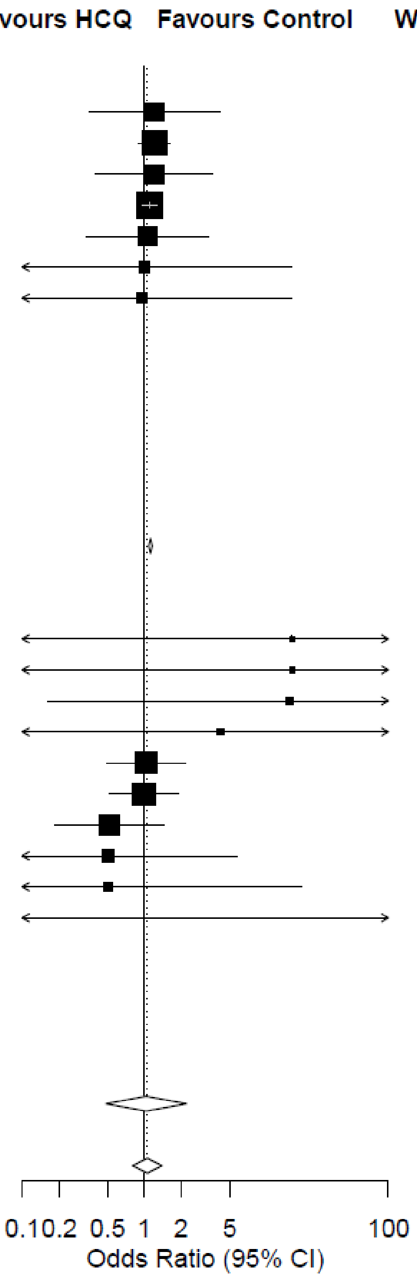

In this meta-analysis we included data from 14 published trials with overall 8981 patients. In these trials, 3809 patients were treated with HCQ, of whom 547 died. 5172 patients were allocated to the control group of whom 893 died. We included data from 12 unpublished trials with 1031 patients. In these trials, 507 patients were treated with HCQ of whom 59 died. 524 were allocated to the control group using placebo of whom 67 died. The dashed vertical line denotes an odds ratio of 1.0, which represents no difference in risk between HCQ and the control. The black horizontal bars represent 95% confidence intervals (CI). Random-effects model of the Hartung-Knapp-Sidik-Jonkman

(HKSJ) approach was performed to obtain a pooled estimate of the odds ratio. The estimate of heterogeneity (τ^2) was obtained using the Sidik-Jonkman (SJ) estimator. We describe the between-trial heterogeneity using the I^2-statistic. The results of the statistical tests for overall effect and corresponding p-values are presented. All tests were two-tailed.

Figure S3B. Forest plot of MH-DSL model. Zero events were corrected by adding the reciprocal of the size of the contrasting study arm.

Source	OR (95% CI)	Weight, %
**Published***		
NCT04353336	1.21 [0.36; 4.12]	0.9
WHO SOLIDARITY	1.21 [0.89; 1.63]	15.2
Coalition I	1.20 [0.40; 3.64]	1.1
RECOVERY	1.11 [0.96; 1.27]	73.7
TEACH	1.07 [0.34; 3.38]	1.1
COVID−PEP	1.01 [0.06; 16.28]	0.2
NO COVID−19	0.96 [0.06; 16.22]	0.2
ChiCTR2000029868		0.0
NCT04261517		0.0
BCN PEP CoV−2 Study		0.0
ChiCTR2000030054		0.0
ChiCTR2000029559		0.0
NCT04491994		0.0
NCT04384380		0.0
**Random subtotal (I^2=0%)**	**1.12 [0.99; 1.27]**	**92.4**
**Not Published**		
OAHU−COVID19	16.32 [0.03; 10108.25]	0.0
COV−HCQ	16.26 [0.01; 25472.06]	0.0
ARCHAIC	15.40 [0.16; 1459.17]	0.1
NCT04335552 A	4.20 [0.04; 440.96]	0.1
REMAP−CAP	1.04 [0.49; 2.18]	2.5
HYDRA	0.99 [0.51; 1.90]	3.2
HYCOVID	0.52 [0.19; 1.45]	1.3
PROTECT B	0.51 [0.04; 5.76]	0.2
NCT04335552 B	0.50 [0.01; 19.56]	0.1
PROTECT A	0.02 [0.00; 100970.20]	0.0
PATCH		0.0
PROTECT C		0.0
CCAP−1		0.0
NCT04333654		0.0
COMIHY		0.0
**Random subtotal (I^2=0%)**	**0.92 [0.60; 1.41]**	**7.6**
**Random effects model**	**1.11 [0.98; 1.24]**	**100.0**

Heterogeneity: I^2 = 0%, τ^2 = 0, p = 0.98
Test for overall effect: p = 0.09

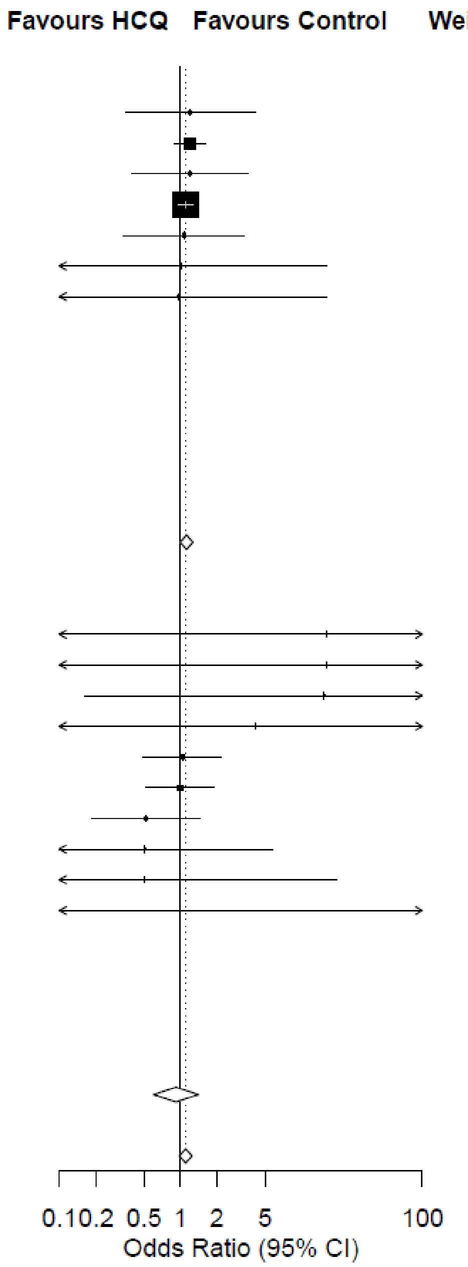

In this meta-analysis we included data from 14 published trials with overall 8981 patients. In these trials, 3809 patients were treated with HCQ, of whom 547 died. 5172 patients were allocated to the control group of whom 893 died. We included data from 12 unpublished trials with 1031 patients. In these trials, 507 patients were treated with HCQ of whom 59 died. 524 were allocated to the control group using placebo of whom 67 died. The dashed vertical line denotes an odds ratio of 1.0, which represents no difference in risk between HCQ and the control. The black horizontal bars represent 95% confidence intervals (CI). Random-effects model of the Mantel-Haenszel (MH) approach was performed to obtain a pooled estimate of the odds ratio. The estimate of heterogeneity (τ^2) was obtained

using the DerSimonian-Laird (DSL) estimator. We describe the between-trial heterogeneity using the I^2-statistic. The results for the statistical tests of overall effect and corresponding p-values are presented. All tests were two-tailed.

Figure S3C. Forest plot of Peto model. Zero events were corrected by adding the reciprocal of the size of the contrasting study arm.

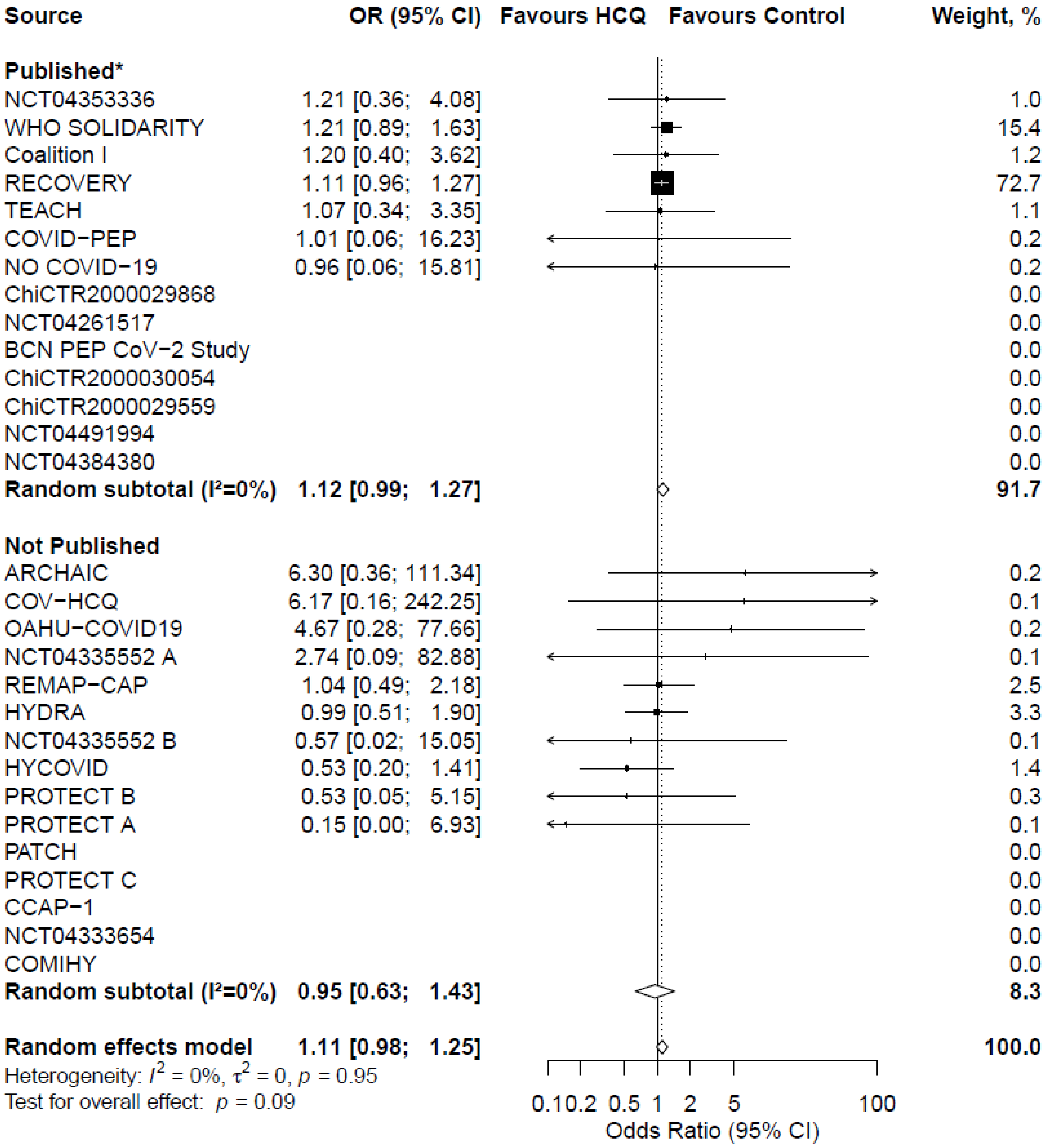

In this meta-analysis we included data from 14 published trials with overall 8981 patients. In these trials, 3809 patients were treated with HCQ, of whom 547 died. 5172 patients were allocated to the control group of whom 893 died. We included data from 12 unpublished trials with 1031 patients. In these trials, 507 patients were treated with HCQ of whom 59 died. 524 were allocated to the control group using placebo of whom 67 died. The dashed vertical line denotes an odds ratio of 1.0, which represents no difference in risk between HCQ and the control. The black horizontal bars represent 95% confidence intervals (CI). Random-effects model of the PETO approach was performed

to obtain a pooled estimate of the odds ratio. The estimate of heterogeneity (τ^2) was obtained using the Paule-Mandel estimator. We describe the between-trial heterogeneity using the I^2-statistic. The results of the statistical tests for the overall effect and corresponding p-values are presented. All tests were two-tailed.
