# Supplementary material for: Mortality outcomes with hydroxychloroquine and chloroquine in COVID-19 from an international collaborative meta-analysis of randomized trials
Source: Nat Commun. 2021 Apr 15;12:2349. doi: 10.1038/s41467-021-22446-z (PMC8050319; doi:10.1038/s41467-021-22446-z)
Supplement: Supplementary file 3 — Reporting Summary [file 41467_2021_22446_MOESM3_ESM.pdf]

nature research

Corresponding author(s): Lars G. Hemkens

Last updated by author(s): Feb 20, 2021

Reporting Summary

Nature Research wishes to improve the reproducibility of the work that we publish. This form provides structure for consistency and transparency in reporting. For further information on Nature Research policies, see our Editorial Policies and the Editorial Policy Checklist.

Statistics

For all statistical analyses, confirm that the following items are present in the figure legend, table legend, main text, or Methods section.

n/a	Confirmed	
☐	☒	The exact sample size (*n*) for each experimental group/condition, given as a discrete number and unit of measurement
☒	☐	A statement on whether measurements were taken from distinct samples or whether the same sample was measured repeatedly
☐	☒	The statistical test(s) used AND whether they are one- or two-sided<br>*Only common tests should be described solely by name; describe more complex techniques in the Methods section.*
☐	☒	A description of all covariates tested
☐	☒	A description of any assumptions or corrections, such as tests of normality and adjustment for multiple comparisons
☐	☒	A full description of the statistical parameters including central tendency (e.g. means) or other basic estimates (e.g. regression coefficient) AND variation (e.g. standard deviation) or associated estimates of uncertainty (e.g. confidence intervals)
☐	☒	For null hypothesis testing, the test statistic (e.g. *F*, *t*, *r*) with confidence intervals, effect sizes, degrees of freedom and *P* value noted<br>*Give P values as exact values whenever suitable.*
☒	☐	For Bayesian analysis, information on the choice of priors and Markov chain Monte Carlo settings
☒	☐	For hierarchical and complex designs, identification of the appropriate level for tests and full reporting of outcomes
☒	☐	Estimates of effect sizes (e.g. Cohen's *d*, Pearson's *r*), indicating how they were calculated

Our web collection on statistics for biologists contains articles on many of the points above.

Software and code

Policy information about availability of computer code

Data collection	For data collection Excel for the Web and Microsoft Excel (Microsoft Office Plus 2019) were used.
Data analysis	The meta-analyses were completed using R version 3.5.1 and the 'meta' package version 4.13-0. The detailed R code can be found on the open science framework: https://osf.io/gehfx/.<br>The Cochrane risk of bias tool (version 2.0) was used for the risk of bias assessment.

For manuscripts utilizing custom algorithms or software that are central to the research but not yet described in published literature, software must be made available to editors and reviewers. We strongly encourage code deposition in a community repository (e.g. GitHub). See the Nature Research guidelines for submitting code & software for further information.

Data

Policy information about availability of data

All manuscripts must include a data availability statement. This statement should provide the following information, where applicable:

- Accession codes, unique identifiers, or web links for publicly available datasets
- A list of figures that have associated raw data
- A description of any restrictions on data availability

All source data generated or analyzed during this study are included in this paper and its supplementary information files. The data files are provided in the Open Science Framework [https://osf.io/qesv4/].

Field-specific reporting

Please select the one below that is the best fit for your research. If you are not sure, read the appropriate sections before making your selection.

- [x] Life sciences
- [ ] Behavioural & social sciences
- [ ] Ecological, evolutionary & environmental sciences

For a reference copy of the document with all sections, see nature.com/documents/nr-reporting-summary-flat.pdf

Life sciences study design

All studies must disclose on these points even when the disclosure is negative.

Sample size	The aim of this meta-analysis was to summarize all available data from trials assessing the effect of hydroxychloroquine on mortality in COVID-19. We had no prespecified hypothesis regarding the possible effect of hydroxychloroquine or a possible effect size that we aimed to test and we did not conduct a sample-size calculation.
Data exclusions	We excluded trials which investigated HCQ and CQ as an preemptive treatment for COVID-19/SARS-CoV-2 infection, because this study focused on the effect of treatment with HCQ or CQ on COVID19 disease. We excluded studies comparing HCQ or CQ with other active treatments, as it would not have been possible to compare the possible risk reduction for death between different active comparators.
Replication	We provide all data and code to replicate the meta-analyses on the open science framework [https://osf.io/qesv4/].
Randomization	This meta-analyses only includes randomized controlled trials.
Blinding	This is a meta-research project and we used prespecified methods for the calculations. There was no risk of bias introduced due to unblinded meta-reserachers. Blinding within the included trials was within the responsibility of the trialists and was considered within the risk of bias assessment.

Reporting for specific materials, systems and methods

We require information from authors about some types of materials, experimental systems and methods used in many studies. Here, indicate whether each material, system or method listed is relevant to your study. If you are not sure if a list item applies to your research, read the appropriate section before selecting a response.

Materials & experimental systems

n/a	Involved in the study	
x		Antibodies
x		Eukaryotic cell lines
x		Palaeontology and archaeology
x		Animals and other organisms
	x	Human research participants
	x	Clinical data
x		Dual use research of concern

Methods

n/a	Involved in the study	
x		ChIP-seq
x		Flow cytometry
x		MRI-based neuroimaging

Human research participants

Policy information about studies involving human research participants

Population characteristics	We considered all clinical trials that randomly allocated patients with confirmed or suspected SARS-CoV-2 infection to a treatment protocol containing HCQ or CQ or the same treatment protocol not containing HCQ or CQ. We did not put any restrictions on age, sex, localisation or comorbidities. All but three trials excluded children and the majority excluded pregnant or breastfeeding women; generalizability remains unclear for those populations
Recruitment	Most patients were recruited within an inpatient setting, while five studies also recruited patients within an outpatient setting. For the meta-analyses itself, no patients were recruited.
Ethics oversight	All unpublished trials were performed according to the principles of the Declaration of Helsinki and written informed consent was obtained from the study participants. Ethical approval was granted by institutional review boards as follows: University of Pennsylvania, ref. #842838 (PATCH, NCT04329923); National Bioethics Committee (NBC) Pakistan, ref. 4-87/NBC-471-COVID-19-05/20/ (PROTECT, NCT04338698); Ethics Committee of the Capital Region of Denmark, ref. H-20025317 (CCAP-1, NCT04345289); Comité de Protection des Personnes du Sud-Ouest et Outre-Mer 4, ref. CPP2020-03-036 / 2020-001271-33 / 20.03.24.72431, and the Agence Nationale de Sécurité du Médicament et des produits de santé (ANSM), ref. MEDAECNAT-2020-03-00045 (HYCOVID, NCT04325893); Ethik-Kommission an der Medizinischen Fakultät der Eberhard-Karls-Universität und am Universitätsklinikum Tübingen, ref. 190/2020AMG1 and ref. 225/2020AMG1 (COV-HCQ, NCT04342221; and COMIHY, NCT04340544, respectively); London-Surrey Borders Research Ethics Committee in the UK, Medisch Ethische Toetsingscommissie Utrecht (METC Utrecht) in the Netherlands, Sydney Local District ethics Review Committee (Royal Prince Alfred Hospital) in Australia, Northern A Health and Disability Ethics Committee in New Zealand, St Vincent's Healthcare Group Ethics and Medical Research Committee in Ireland, King Abdullah International Medical Research Center Institutional Review Board in Saudi Arabia, University of Pittsburgh Institutional Review Board in the United States, Unity Health Research Ethics Board in Canada, National Ethics Committee for Clinical Research (CEIC) in Portugal, and the Romania Academy of Medical Sciences National Bioethics Committee for Medicines and Medical Devices (REMAP-CAP, NCT02735707); Comissão Nacional de Ética em Pesquisa (CONEP), ref. 3.961.681 (CloroCOVID19II A, NCT04323527, and CloroCOVID19II B, NCT04342650); a Single Ethics Committee from the Coordination of the National Institutes of Health and High Specialty Hospitals, ref. C13-20 (HYDRA, NCT04315896); the Ethics Committee of Beijing Youan Hospital,Capital Medical University, ref. JINYOUKELUN[2020]013 (ChiCTR2000031204); Partners Human Research Committee at the Brigham and Women's Hospital, Boston, Ethisch Comite in Belgium, and Stichting Beoordeling Ethiek Biomedisch in the Netherlands (NCT04333654); The Queen's Medical Center, ref. RA-2020-018 (OAHU-COVID19, NCT04345692); Duke University Medical Center Institutional Review Board, ref. Pro00105339, and UnityPoint Health Institutional Review Board (NCT04335552); and Medical Ethics Committee Utrecht (METC Utrecht), part of the Dutch Central Committee on Research Involving Human Subjects (ARCHAIC, NL8490).

Note that full information on the approval of the study protocol must also be provided in the manuscript.

Clinical data

Policy information about clinical studies

All manuscripts should comply with the ICMJEguidelines for publication of clinical research and a completedCONSORT checklist must be included with all submissions.

Clinical trial registration	The study protocol was published on the open science framework: https://osf.io/qesv4/.
Study protocol	The study protocol was published on the open science framework: https://osf.io/qesv4/.
Data collection	We used no individual patient data. All data that we used were aggregated, anonymous, trial information. We searched for eligible trials registered at ClinicalTrials.gov and the WHO International Clinical Trials Registry Platform (ICTRP) by June 11, 2020 (COVID-evidence database, covid-evidence.org). We additionally searched PubMed and the Cochrane COVID-19 trial registry (covering preprints, trial registries and literature databases) by June 11, 2020, using terms related to HCQ and CQ combined with terms for COVID-19 and a standard RCT filter.<br>We obtained aggregated, anonymous trial-level data from non-published trials from clinical trial study groups directly who are co-authors of this study. We updated the literature search on October 16, 2020.
Outcomes	All-cause mortality
